# Improved Setup for Decolourization Experiments with Granular and Powdered Adsorbent Materials Using UV-VIS Flow-through Cells

**DOI:** 10.1016/j.mex.2025.103289

**Published:** 2025-03-26

**Authors:** Martin Behringer, Harald Hilbig, Brigitte Helmreich, Alisa Machner

**Affiliations:** a Technical University of Munich, TUM School of Engineering and Design, Professorship for Mineral Construction Materials, Lichtenbergstr. 2, 85748 Garching, Germany; b Technical University of Munich, TUM School of Engineering and Design, Chair of Urban Water Systems Engineering, Am Coulombwall 3, 85748 Garching, Germany

**Keywords:** Method development, Textile wastewater treatment, Online detection, Decolourization setup using UV–VIS flow through cells

## Abstract

Textile wastewater treatment poses global challenges due to complex and costly processes, particularly in the adsorption-based decolourization step. Existing experimental methodologies for adsorption suffer from inconsistencies, hindering comparability across studies. To address this, we developed a universal setup integrating conventional adsorption methods with pharmaceutical dissolution techniques. This approach provides continuous UV-VIS monitoring of adsorption processes without external filtration, which is suitable for both fine powders (∼microns) and granular particles (∼millimetres) and is applicable to both natural and synthetic adsorbents. Case studies conducted with powdered and granular adsorbents confirmed this method's robustness, reproducibility, and enhanced accuracy, allowing real-time, precise monitoring. Overall, this versatile approach significantly improves reliability in adsorption experiments, offering a broadly applicable solution for adsorption monitoring in wastewater treatment research.

• A versatile setup combining adsorption methods with flow-through UV-VIS spectrometry.
• Enables continuous monitoring of decolourization without the need for external filtration.
• Applicable to a wide range of adsorbent materials, from fine powders to granulates.

## Graphical abstract

**Figure:**
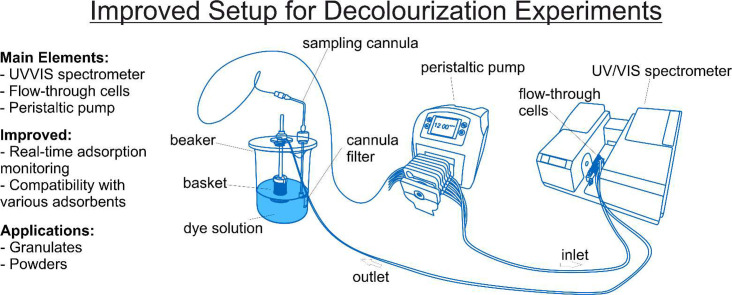

Specifications table

**Table:** 

Subject area:	Environmental Science
More specific subject area:	Wastewater treatment
Name of your method:	Decolourization setup using UV–VIS flow through cells
Name and reference of original method:	M. Wagner, C. Eicheler, B. Helmreich, H. Hilbig, D. Heinz, Removal of Congo Red From Aqueous Solutions at Hardened Cement Paste Surfaces, Front. Mater. 7 (2020). doi: 10.3389/fmats.2020.567130
Resource availability:	Any UV-VIS spectrometer with flow-through cells

## Background

The rapid expansion of fast-fashion industries has led to a significant increase in textile wastewater, posing global challenges in its treatment due to complex, high-cost processes. Adsorption-based decolourization is a critical step in wastewater treatment, and low-cost adsorbents are increasingly explored for this purpose due to their economic feasibility. The adsorption capacity of these materials serves as a key indicator of their efficacy. However, experimental methodologies for evaluating adsorption in batch studies vary widely—ranging from magnetic stirrers with periodic sample collection [1,2] to agitated Erlenmeyer flasks [3] and multishaker vessels [4]. Most existing research focuses on investigating the adsorption properties of specific adsorbents. However, to the authors’ best knowledge, no study has yet presented a robust, reproducible experimental method that is not narrowly tailored to particular adsorbent characteristics. Although several review articles compare low-cost adsorbents [5,6], they do not focus on the methodological differences.

These inconsistencies hinder comparisons of adsorption capacities across studies and introduce questions about measurement accuracy. Additionally, since analyses are often performed at various time steps and involve filtration steps, comparability becomes difficult, as filtration can introduce artefacts, which can alter the measurement [7].

To address these challenges, our study introduces a universally applicable approach that integrates conventional batch adsorption techniques with components from pharmaceutical dissolution testing [8]. This setup enables continuous UV-VIS monitoring of adsorption in materials ranging from fine powders (∼microns) to granular particles (∼millimetres) without external filtration, thus improving measurement accuracy. Employing hydrotalcite powder and hydrated cementitious granules as case studies provides a reproducible and detailed procedure for assessing decolourization capabilities across diverse adsorbent materials, facilitating more reliable and comparable adsorption evaluations.

This method introduces a unique approach that combines batch adsorption with pharmaceutical dissolution testing, enabling continuous UV–VIS monitoring without external filtration or centrifugation.

## Materials and equipment

This method suits two types of adsorbent materials: fine powders and granular materials. For demonstration purposes, we utilised hydrotalcite powder and ground hydrated cement paste, representing distinct forms of adsorbents commonly employed in dye decolourization studies [6].

Hydrotalcite powder was synthesised following the procedure proposed by Feitknecht & Fischer [9,10]. Post-synthesis, the hydrotalcite was dried in a vacuum oven at approximately 200 hPa and 120 °C for 20 h. The dried material was then finely ground using a mortar mill with maximum pestle pressure and subsequently sieved to <63 µm, resulting in a mean particle size (d₅₀) of 11.2 µm. This fine powder serves as an example of a microparticulate adsorbent that is effective for dye decolourization applications [11].

Hydrated cement paste granulates were prepared by mixing Ordinary Portland Cement (OPC) with distilled water at a weight ratio of 1:0.5 (water to cement). The mixture was cast into prism moulds with dimensions of 4 cm × 4 cm × 16 cm and demoulded after 24 h. The prisms were then stored in a 1 M Ca(OH)₂ solution for one month to ensure the paste was well-hydrated. Following this period, the prisms were slowly dried at 20 °C in air and transferred to closed plastic containers. After drying, the material was crushed using a jaw crusher and sieved to obtain granules ranging from 2 to 4 mm. These granulates were further stored in closed plastic boxes for approximately one year until further measurements. The hydrated cement paste consists of several distinct phases that not only can adsorb dyes but also can release other compounds into the wastewater [11].

All adsorption experiments employed the anionic dye Reactive Blue 19, selected for its prevalent use in textile wastewater treatment studies [[11], [12], [13]]. Reactive Blue 19 (Sigma Aldrich, CAS-No. 2580–78–1) was dissolved in distilled water to prepare a stock solution with a concentration of 100 mg/L. This dye solution ensured consistency and reliability across all decolourization assessments. Recognising that pH significantly affects adsorption behaviour [6], we did not include pH control measures in our experiments.

**General equipment**

**Table:** 

UV–VIS spectrometer:	Perkin-Elmer Lambda 465 UV–VIS
Flow-through cells:	8-channel flow-through cells; optical path length = 10 mm
Peristaltic pump:	ALS PCP 151 L Metering Pump
Pump tubing:	3-stop pharmaprene tubing; 1.52 mm ID
Connector tubing:	PTFE tubing; 1 mm ID; 1.6 mm OD; ¼ - 28 Fittings on both ends
Tubing adapters:	Stainless steel Luer adapter
	Screw-in connectors (1/4″−28)

**Batch equipment**

**Table:** 

Beaker:	800 mL, low form
Basket:	Riggtek stationary basket assembly with shaft and 40 mesh basket; 316 stainless steel
Cannula:	Riggtek sampling cannulae; 195 mm; 3.2 mm OD; bent; stainless steel adapter
Cannula stopper:	3.6 mm; for covers with 1.35 cm hole; self-tightening
Cannula filter:	ERWEKA 10 µm cannula filters; UHMW polyethylene; 3.2 mm ID

### Experimental setup and configuration

**Fig. 1:**
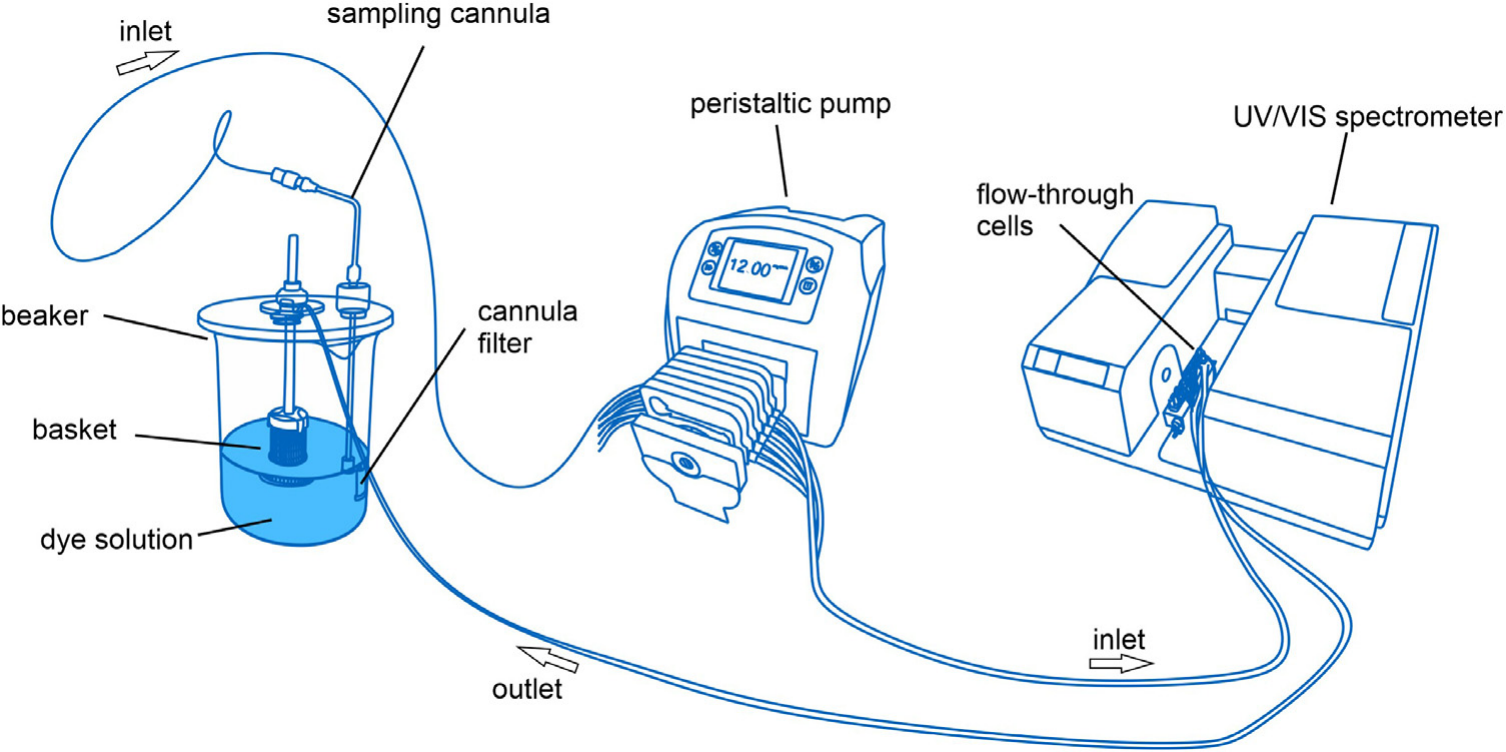
Experimental Setup: beaker with a sampling basket and sampling cannula (left), peristaltic pump (middle), and UV-VIS spectrometer (right).

The experimental setup comprises a UV–VIS spectrometer, a pump, and a container for adsorbents and dye solution, as shown in Fig. 1. The UV–VIS spectrometer, equipped with an 8-channel flow-through cell, represents the central component of the setup. While not essential to the method, an automated sample changer unit facilitates sequential analysis of multiple samples without manual intervention. This allows for measurements of up to eight batches at the same time.

**Fig. 2:**
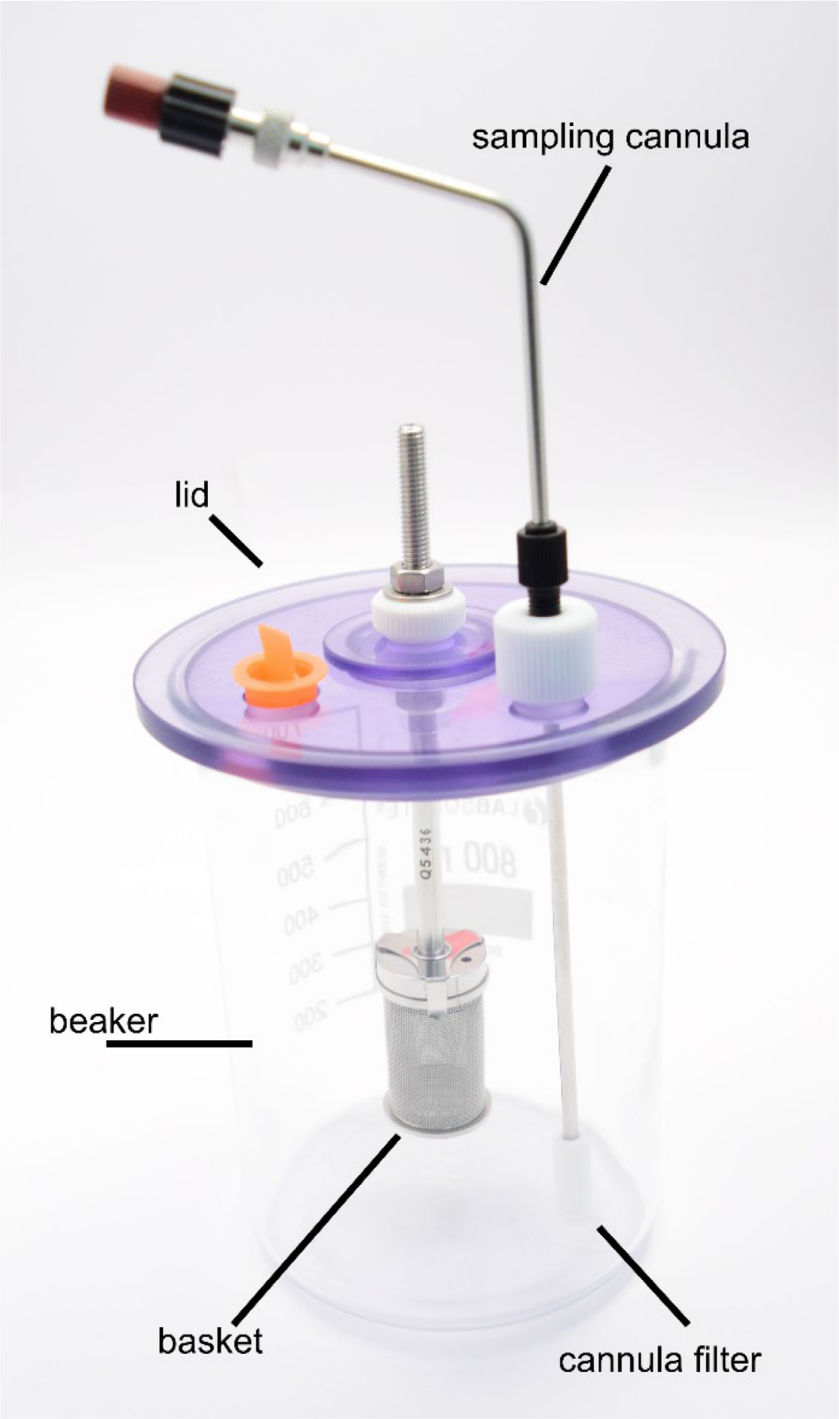
General decolourization setup consisting of an 800 mL beaker (low-form), a vessel cover, a sampling tube equipped with a filter, and the sampling basket (40 mesh).

The spectrometer is connected to the metering pump, which is controlled via the software IDISis (Automated Lab Systems, UK). The software facilitates the synchronisation of the pump and spectrometer operations, enabling seamless control and data acquisition. The spectrometer and pump are linked using bundled PTFE tubing, which ensures chemical resistance and maintains system integrity under various flow conditions. This configuration establishes a closed-loop system in which the dye solution continuously circulates through the flow-through cells, enabling real-time monitoring of the decolourization process. The reaction container consists of an 800 mL low-form beaker, which is the primary vessel for the adsorbents and dye solution. A vessel cover is secured atop the beaker, featuring a sampling basket (cf. Fig. 2). This configuration prevents particle contamination during UV–VIS analysis by containing the adsorbents while allowing the dye solution to circulate freely. Moreover, the vessel cover includes a second opening that permits the return flow of the solution, ensuring uninterrupted circulation within the closed-loop system. The beaker is positioned on a magnetic stirring plate set to a specific stirring speed (e.g., 300 rpm) to guarantee uniform mixing of the adsorbents and dye solution.

### Batch experiment setup

In evaluating the decolourization capabilities of diverse powdered adsorbents, a principal challenge is the prevention of solid particles from contaminating the UV-VIS analysis chamber. The sampling cannulas were, therefore, equipped with filters that were utilised to address this issue. The filters should be selected based on their chemical resistance, filtration efficiency, and compatibility with the dye solution. Before the adsorption experiments, preliminary tests were performed to ensure the filters did not adsorb the reactive dye. This was done to guarantee that only the dye solution reached the UV-VIS spectrometer. These filters effectively captured particulate matter, maintaining the integrity of the absorbance measurements.

The filter size can be adjusted according to the mean particle size of the adsorbent being tested by replacing the cannula with one fitted with an appropriate mesh size. This adjustment prevents clogging while allowing sufficient flow, ensuring consistent and reliable measurements across different adsorbent types.

During the experiment, the dye solution is drawn into the pump through the sampling cannula equipped with the filter and then pumped through the UV-VIS sample cell. A full spectrum scan is conducted within the spectrometer, and the dye solution is returned to the beaker, allowing for continuous monitoring of decolourization. This setup ensures real-time measurement of dye concentration, facilitating accurate assessment of the adsorbent's decolourization capabilities.

The experimental setup employed to test granulates differs slightly from that used for powders. To prevent the granulates from being ground by the magnetic stirrer, they are placed in a sampling basket with a mesh size adapted to their particle size. Stainless steel baskets were used to ensure chemical resistance and durability. This configuration permits the dye solution to interact with the granulate surfaces without causing the granulates to disintegrate due to contact with the magnetic stirrer during the experiment. The vessel cover incorporates an opening for the sampling cannula equipped with its respective filter, as well as a supplementary opening to facilitate the return flow of the solution to the beaker. This setup enables continuous monitoring of the decolourization process without compromising the physical structure of the granulates.

### Experimental procedure

Before measurements, the UV-VIS spectrometer was turned on and allowed to stabilise for a few minutes. A blank measurement was performed using deionised water in the first cell to remove background signals from non-analyte components. Subsequently, a baseline measurement was conducted across all cells with deionised water to account for variations in the optical path.

After completing the blank and baseline measurements, the cells were emptied and filled with the dye solution to begin the experimental procedure. It was ensured that the dye was dissolved entirely prior to the first measurement. The initial measurement was performed without the adsorbent present, setting the baseline at 100 % dye concentration to correct for minor weighing fluctuations.

Since adsorption occurs almost immediately upon adding the adsorbent, the first ten measurements were taken at one-minute intervals, followed by ten measurements every five minutes and twelve measurements every ten minutes. The measurement intervals are limited only by the time required for each measurement, which ranges from 5 to 20 s per cell. The pump was operated for one minute before each measurement to ensure the liquid was entirely replaced between scans. This slightly delays the measurements at the beginning, resulting in a systematic delay of the reading times with short reading intervals. Continuous pumping during measurement eliminates this delay but can potentially affect the measurement. The short intervals were explicitly selected to optimally track the fast adsorption kinetics in the early phase, with the intervals gradually increasing as the adsorption slowed down over time.

The adsorbent powder was added immediately after the initial reading by pouring it through the opening into the beaker. This gives the first reading a c/c_0_ of precisely 1, allowing buffer weighing fluctuations. The cannula filters prevent the solid particles from entering the analysis chamber. The experiment was then run for the desired duration, continuously monitoring the decolourization process.

The procedure for measuring the adsorption capacity of granulates is similar to that for powders, with slight modifications to accommodate their larger size and prevent physical disintegration. Instead of pouring the powder directly into the liquid, the granulates were weighed and placed into a sampling basket with an appropriate mesh size before the measurement. Once the initial measurement was taken, the basket was lowered into the beaker, allowing the dye solution to flow uniformly through the granulates. Furthermore, the cannula filter prevents small particles from entering the analysis chamber, maintaining measurement accuracy.

### Data evaluation

**Fig. 3:**
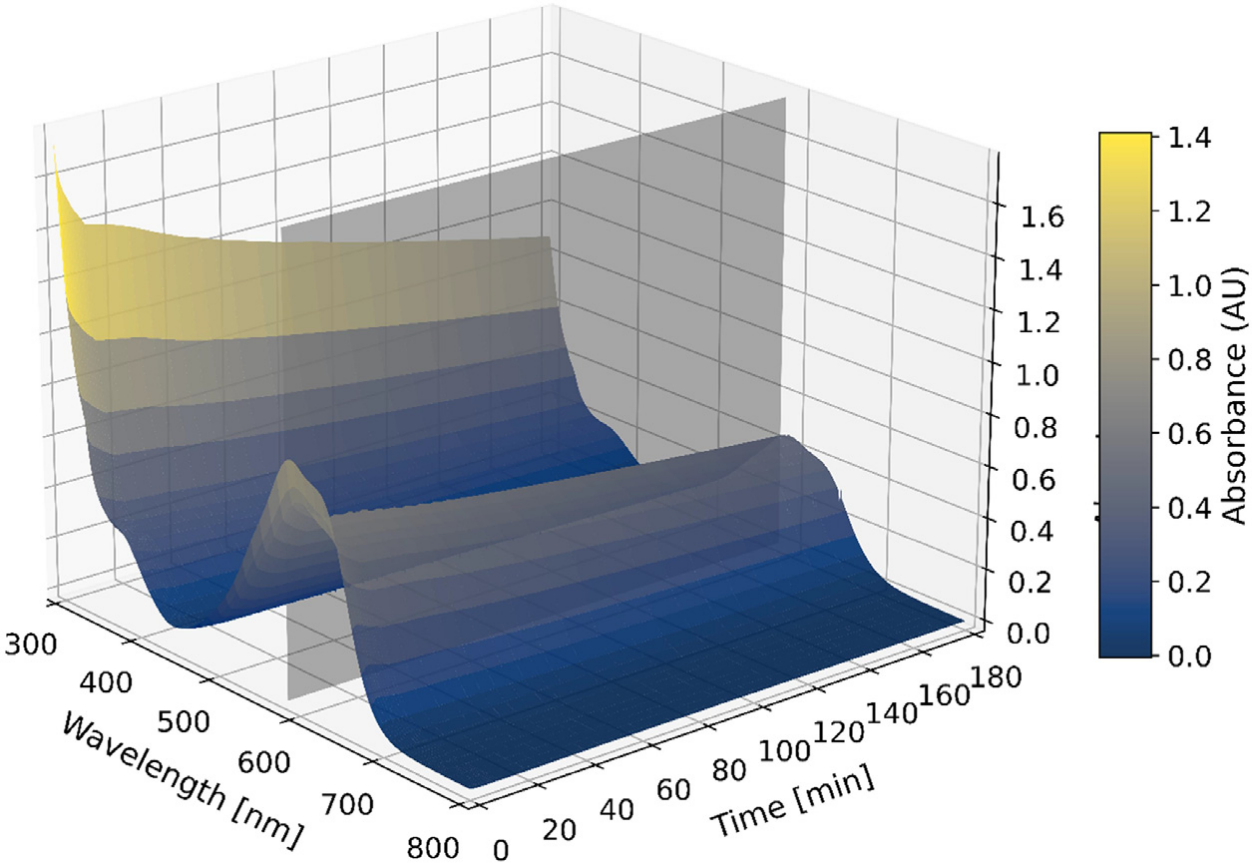
Three-dimensional representation of the measured absorbance data as a function of wavelength, time, and absorbance for cementitious granulates. The grey plane indicates the selected wavelength for further analysis.

**Fig. 4:**
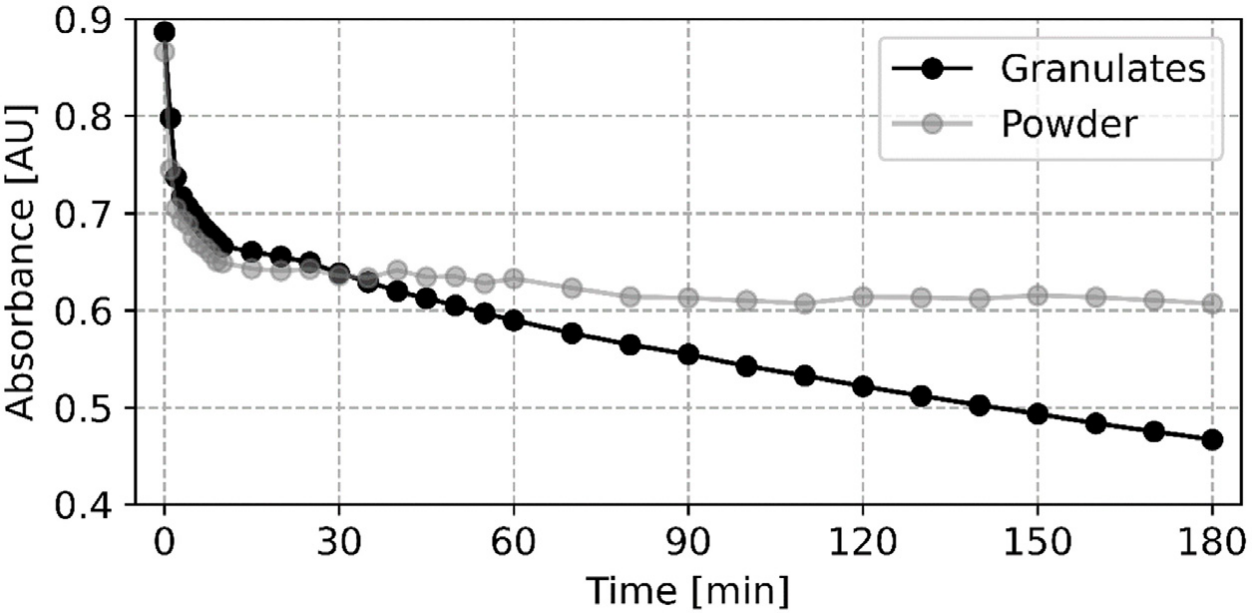
Two-dimensional representation of absorbance overtime at 585 nm for a 3-hour adsorption experiment, comparing granulates (intersection of grey plane and data points from Fig. 3) and powder.

The measured data can then be analysed. Fig. 3 visually represents the data points recorded during an example 3-hour measurement, capturing absorbance across a range of wavelengths over time. In this example, one specific wavelength – indicated by the grey plane – was selected to represent the data in a two-dimensional format by plotting absorbance versus time, as illustrated in Fig. 4. Fig. 3 also enables a rapid scan of changes in the absorption spectra at specific time points, which may indicate chemisorption or other reactions occurring in the solution (e.g., pH-dependent dye behaviour).

Data was evaluated using Python (version 3.12.4, libraries such as NumPy, Pandas, and Matplotlib), Microsoft Excel, and OriginPro. Data visualisation was created using Python. The absorbance data, representing the measured light absorption over time for each wavelength within the selected range, was initially corrected for outliers. The outlier correction was done based on an adaptive local threshold, whereby measurements exceeding 1.5 times the median of neighbouring values were identified as outliers and excluded from further analysis. Following outlier correction, the relative concentration (c/c₀) [-] was calculated for each measurement, where c [mg/L] represents the dye concentration at time t [min] and c₀ [mg/L] is the initial dye concentration measured before the addition of adsorbent. c₀ was determined from the first measurement after the blank and baseline calibrations, where no adsorbent was present. This normalisation facilitates a precise analysis of the adsorption kinetics throughout the experiment by providing a standardised reference point. Data visualisation (Figs. 3 and 4) was achieved using Python and OriginPro, allowing for the creation of adsorption isotherms and kinetic plots to interpret the adsorption behaviour of different materials under varying experimental conditions.

### Adaptations

This configuration has been designed with versatility, allowing batch and column experiment configurations. Column experiments are essential in research on textile wastewater treatment as they closely mimic real-world continuous flow conditions, providing insights into the scalability and adsorption kinetics.

In the experimental setup, the identical tubing and spectrometer configuration is adapted to facilitate the passage of the dye solution through a packed column containing granulated adsorbents, thereby enabling continuous flow-based decolourization analysis at a controlled flow rate.

**Fig. 5:**
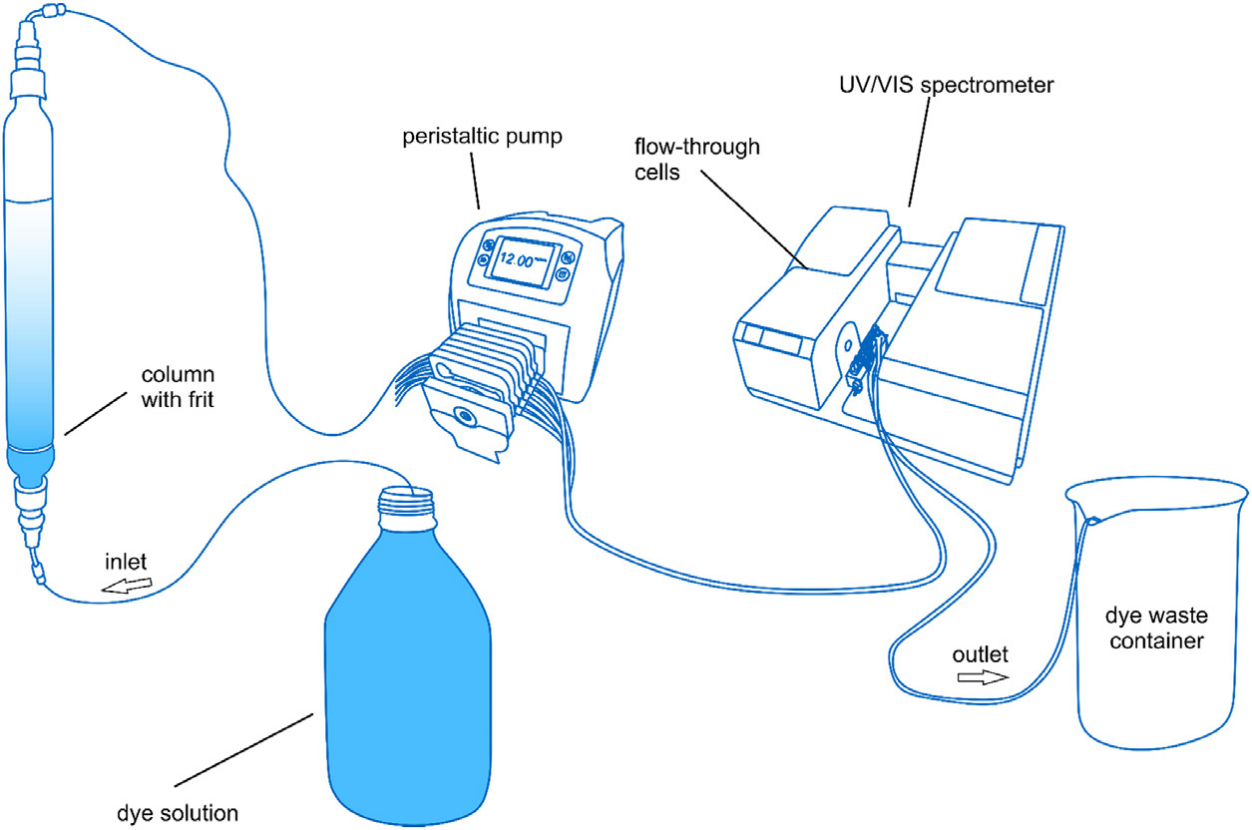
Experimental setup for column experiments.

The setup used for columns was configured as an open system (cf. Fig. 5), although it can be modified into a closed-loop system with minor adjustments if necessary. The setup included a container for the dye solution connected via PTFE tubing to the pump. In our experiment, the solution was pumped from the bottom to the top because the coarse granulates could have allowed the dye solution to bypass the adsorbent if pumped from the top down due to edge effects.

The column, fitted with a frit, was loaded with granulates and connected to the UV-VIS spectrometer. After measurement, the solution was directed into a waste container. In this method, we describe only the latter approach without recirculation.

After calibration, the dye solution was pumped into the columns at a controlled flow rate. The column was filled with the adsorbent but not pre-filled with liquid before the experiment started to prevent premature interaction between the dye and adsorbent. The first measurement was taken when the dye solution reached the frit at the top of the column. Depending on the size of the column, it may take minutes to hours for the dye solution to reach the measurement cell for the first time. Therefore, the measurement times cannot be as tightly scheduled as for the powders and granulates and should be adjusted accordingly to guarantee sufficient resolution. Data handling and evaluation for the column experiments were conducted in the same manner as described for the batch experiments.

### Limitations

This method necessitates using a UV-VIS spectrometer equipped with one or multiple flow-through cells. While additional equipment, such as automated software to control the pump during measurements, can significantly enhance the efficiency and throughput of the method, it is not strictly essential. The core procedure can be conducted with basic laboratory equipment and manual control, thereby making the method accessible to a wide range of laboratories with varying resource levels.

Numerous factors can influence adsorption measurements, and users must be aware of these potential effects. In particular, mixing parameters significantly impact the measured adsorption. Our validation experiments revealed variations of up to 4 % in absorbance (c/c₀) when using magnetic stirrers of different sizes (ranging from 2 to 4 cm in length, with 3 cm as reference) and up to 10 % when employing different stirring speeds (ranging from 100 to 500 rpm, with 300 rpm as reference).

Additionally, careful selection and maintenance of filters and mesh sizes are necessary to prevent clogging and ensure accurate measurements. While alternative filtration methods can be employed based on available resources, improper filtration can lead to particulate contamination, compromising absorbance data integrity. The method is sensitive to operational parameters such as stirring speed and pump flow rate, which must be carefully chosen and maintained to obtain consistent results.

Some materials may limit the effective use of high-resolution, early-stage adsorption monitoring due to transient peaks or rapid adsorption kinetics [11]. In such cases, adjustments to the measurement intervals or initial setup may be necessary to capture the rapid changes accurately without oversampling or missing critical adsorption phases. Additionally, very fine natural adsorbents can limit the method as the cannula filter is limited to a specific minimum pore size, which still lets water penetrate. These very fine adsorbents, such as clay, can clog the filter and impede consistent water flow.

This method offers a unique and universally applicable approach to adsorption measurements across different materials and experimental conditions, provided flow-through cells are available.

## Conclusion

In conclusion, the presented adsorption setup demonstrates a universal, robust, and reproducible method for real-time dye decolourization monitoring in textile wastewater treatment. Integrating continuous UV-VIS measurements with adaptable filtration strategies effectively accommodates both fine powders and coarse granulates, eliminating the need for external filtration steps. This approach resolves the inconsistencies prevalent in traditional batch studies, enabling more accurate and comparable adsorption data. Moreover, the inclusion of column experiments extends its applicability to continuous-flow conditions, bridging laboratory studies and real-world scenarios. Adoption of this versatile setup thus has the potential to standardise adsorption experiments and significantly advance research efforts in wastewater treatment.

## Ethics statements

Not applicable.

## Declaration of generative AI and AI-assisted technologies in the writing process

While preparing this work, the authors used Grammarly Premium, DeepL Write, and ChatGPT (o1 & o3-mini) to enhance readability and language. After using these tools, the authors reviewed and edited the content as needed and take full responsibility for the content of the published article.

## CRediT authorship contribution statement

**Martin Behringer:** Conceptualization, Methodology, Validation, Formal analysis, Investigation, Data curation, Writing – original draft, Visualization. **Harald Hilbig:** Conceptualization, Methodology, Validation, Writing – review & editing, Funding acquisition, Supervision. **Brigitte Helmreich:** Conceptualization, Methodology, Validation, Writing – review & editing, Funding acquisition, Supervision. **Alisa Machner:** Conceptualization, Methodology, Validation, Writing – review & editing, Supervision.

## Declaration of competing interest

The authors declare that they have no known competing financial interests or personal relationships that could have appeared to influence the work reported in this paper.

## Data Availability

Data will be made available on request.
